# Editorial: Innovative therapies and novel challenges in pediatric intestinal failure

**DOI:** 10.3389/fnut.2022.1050004

**Published:** 2022-12-13

**Authors:** Jutta Köglmeier, Barbara De Koning, Lorenzo Norsa

**Affiliations:** ^1^Department of Paediatric Gastroenterology, Great Ormond Street Hospital for Children NHS Foundation Trust, London, United Kingdom; ^2^Department of Paediatric Gastroenterology, Erasmus MC-Sophia Children's Hospital, Rotterdam, Netherlands; ^3^Department of Pediatric Hepatology, Gastroenterology and Transplantation, ASST Papa Giovanni XXIIII, Bergamo, Italy

**Keywords:** intestinal failure, challenges, novel therapies, children, parenteral nutrition, complications

Intestinal failure (IF) is a serious condition leading to the need for parenteral nutrition (PN). Leading causes in childhood include short bowel syndrome, severe motility disturbances and congenital defects of enterocyte development (1). Although the numbers of children requiring prolonged intravenous nutrition are much smaller compared to adult figures, pediatric IF is a distinct entity which differs from adult disease both in complexity and level of care required.

Whilst far from being a panacea, home PN offers improved quality of life and allows for timely discharge of children who are expected to require intravenous nutrition support for a prolonged or indefinite period (2).

The outcome for children dependent on PN has been transformed within the last 10 years due to the co-ordination of complex care in specialist multidisciplinary nutrition support teams.

As these patients grow older and develop into adulthood whilst continuing PN support, new insight into complications of this chronic condition emerge.

This Research Topic is aimed at collecting papers to increase knowledge and understanding of challenges faced when caring for children with IF and novel therapeutic options available.

Reduction in septic episodes and long term central venous catheter preservation has been achieved with the introduction of anti-septic line locks. Taurolidine, a derivate of the aminoacid taurine, has created particular interest, as not only able to prevent biofilm formation within the lumen of the catheter, but has also direct antimicrobrial effects. No serious systemic side effects have been reported and bacterial resistance against Taurolidine has never been observed (3).

Together with the use of anti-coagulant therapy to prevent (recurrent) clot blood formation in and around the central venous line, catheter losses are reduced in number. With the involvement of dedicated radiologists new techniques have emerged to gain new central venous line access in complex cases with reduced central access site. The importance of a collaboration between surgeons and interventional radiologists are described in this issue discussing advanced techniques.

Contemorary PN composition and IF management prevents the development of cholestasis and IF associated liver disease. The role of the dietitian has been increasingly recognized in the prevention of macro- and micronutrient dieficiencies.

Regular follow up has given insight into more subtile complications of long term PN.

Eating disorders can occur as a consequence of too restrictive diets commonly used in the past. In recent years this approach has changed, and children are encouraged to eat by mouth and learn to appreciate different tastes and textures, rather than being weaned of PN quickly with aggressive tube feeding (4).

Many home PN patients are troubled by reduced levels of energy. Not only the children but also their parents report feelings of stress and anxiety. Early and sustained psychosocial support for these patients and their care givers is essential throughout childhood into adulthood (5, 6).

Whilst adapting strategies to avoid long term complications are important, achieving enteral autonomy remains a key goal of IF rehabiliation. However, biological markers guiding the clinician when and how to transition patients to enteral/oral feeding are lacking. Much of this remains subject to trial and error by increasing the amount of enteral feeding in order to assess the child's tolerance. To date there is no uniform consensus on the optimal type of feed and feeding regime used, and great variability remains amongst centers.

One of the biggest breakthroughs in the management of IF in the latest decade is the approval of the use of glucagon-like peptide-2 (GLP-2) analogs (Teduglutide) in pediatric patients (ages 1–17 years old) in 2016 by the European Medicines Agency. Teduglutide treatment aims to increase intestinal sufficiency by stimulating mucosal hyperplasia (7). In the current Research Topic, we provide the first systematic review on the use of Teduglutide in children. After an accurate literature review 14 articles were found eligible with a total of 223 treated children enrolled. While safety and efficacy seem well demonstrated in the review, a need for studies on long term complications are needed. In relation to this, the Research Topic presents a case report on intestinal mucosal modification in a child 18 months after Teduglutide introduction and weaning from parenteral nutrition. Teduglutide-induced mucosal hyperplasia did not seem to rise concern for malignant degeneration in this particular patient, suggesting that these benign features need confirmation in bigger cohort studies.

Serious complications mentioned above may lead to the transformation from intestinal failure to nutritional failure (8) with subsequent need for intestinal transplantation due to the inability to feed. Intestinal transplantation is still associated with a high rate of graft loss and mortality, mainly due to rejection complication (9). A comprehensive review by Dogra and Hind, provides an interesting general overview on molecular mechanism of intestinal immune cell modulation and introduction innovative drug therapies, microbiome manipulation and tissue engineering to try to overcome intestinal transplantation rejection.

In summary, the papers discussed in this special edition provide new insight into the current state of the art management of IF in childhood and highlight the need for ongoing research to continue to improve the care of this complex disorder.

## Author contributions

JK wrote the introduction and conclusion and edited the final version of the editorial submitted. BD wrote the middle section. LN wrote the final paragraph. All authors contributed to the article and approved the submitted version.
